# Laryngostroboscopy and voice evaluation in adult patients with Parkinson’s disease^☆^^☆☆^

**DOI:** 10.1016/j.bjorl.2025.101620

**Published:** 2025-05-27

**Authors:** Andréa de Carvalho Anacleto Ferrari Castro, Rogério Aparecido Dedivitis, Débora dos Santos Queija, Mario Augusto Ferrari de Castro, Juliana Godoy Portas, Alessandra Sampaio Ferreira

**Affiliations:** Universidade de São Paulo (USP), Faculdade de Medicina, Hospital das Clínicas, Departamento de Cirurgia de Cabeça e Pescoço, São Paulo, SP, Brazil

**Keywords:** Parkinson’s disease, Voice disorders, Voice quality, Laryngoscopy, Speech perception, Speech acoustics

## Abstract

**Objective:**

To evaluate the laryngeal manifestations of PD.

**Methods:**

Evaluation of 18 PD patients conducted between January 2011 and December 2012. Patients were evaluated through videolaryngostroboscopy, auditory perceptual analysis of the voice using the GIRBAS scale, acoustic voice analysis, and application of the Vocal Handicap Index (VHI) questionnaire.

**Results:**

Dysphonia and vocal fatigue were referred by 72.22% and 33.3% of the patients, respectively, whereas changes during laryngoscopy were verified in 94.44%. The medians of the acoustic voice analysis and the VHI self-assessment questionnaire were unchanged. The median of the VHI emotional domain was higher among patients who presented open phase closure, with statistically significant difference (p = 0.029).

**Conclusion:**

Patients with PD present laryngeal and voice changes.

**Level of evidence:**

Level IV.

## Highlights

• Dysphonia and vocal fatigue were referred by 72.22% and 33.3% of the patients.
• Changes during laryngoscopy were verified in 94.44%.
• The medians of the acoustic voice analysis and the VHI self-assessment questionnaire were unchanged.
• The median of the VHI emotional domain was higher among patients who presented open phase closure, with statistically significant difference (p = 0.029).

## Introduction

Parkinson’s Disease (PD) is a chronic, progressive condition with slowly developing symptoms. Tongue, larynx and pharynx disorders are observed in 70%–92% of patients.^1^ Glottic resistance, airflow increase, low subglottic pressure, and decreased loudness are often found in PD.^2^ Vocal changes can be assigned to incomplete glottic closure, reduction in synergy and activation of the laryngeal muscles, muscle atrophy or fatigue, tension or vocal fold movement asymmetry, and rigidity of vocal folds or respiratory muscles.^3^ Stroboscopy can show significant laryngeal abnormalities, such as abnormal adduction and abduction, vocal fold bilateral atrophy, and phase asymmetry.^4^

Lack of further information on this theme and disagreement between some studies have motivated the interest in evaluating this group of patients through stroboscopy and perceptual, acoustic and self-perceptual voice analysis. Thus, this study aimed to evaluate laryngeal and voice changes in adult PD patients.

## Methods

This study was approved by the Institutional Review Board of Centro Universitário Lusíada under protocol nº 120/2011. Eighteen PD patients were prospectively evaluated from January 2011 to December 2012. PD diagnosis was based on the United Kingdom Parkinson’s Disease Society Brain Bank (UKPDSBB) criteria.^5^ The Hoehn and Yahr (HY) Scale was applied to rate individual patient disability.^6^ This scale comprises five classification stages that evaluate PD severity. The patients included in this study presented a score >1.5.

The study sample was composed of 11 men and seven women aged 41–78 years (mean age of 68.2 years). Duration of clinical complaints varied from 3 months to 22 years (mean of 6.2 years). All patients were under treatment with PD drugs. Disease staging varied from 1.5 to 5, and stage distribution of the study participants (stage ‒ number of individuals) was as follows: stage 1.5–4 (21.05%); stage 2.0–3 (15.8%); stage 2.5–5 (26.31%); stage 3.0–4 (21.05%); stage 4.0‒1 (5.26%); and stage 5.0‒1 (5.26%).

All patients underwent videolaryngostroboscopy using a 70 ° rigid Karl Storz® telescope and a Kay Pentax® RLS 9100B rhino-laryngeal stroboscope. The data recorded on DVD were simultaneously and consensually analyzed by three specialists with experience in stroboscopy. A stroboscopic evaluation protocol was also applied considering the following aspects: irregularity of voice fold edge, glottic closure, prevalence of the glottic cycle phase, vertical level, amplitude, mucosal wave, phase symmetry, periodicity, arytenoid movement and symmetry of movement, hyperadduction, site of vibration, mucosal appearance, and mucus formation.^7^

In order to obtain the perceptual computerized evaluation of the patients’ voices, voice samples consisting of sustained phonation of the /a/ vowel at normal intensity and frequency, counting numbers from 1 to 10, and reading of a connected speech passage were recorded using a professional microphone (Shure PG48). The computerized analysis was performed using VoxMetria® 4.4 software. The acoustic parameters fundamental Frequency (F0), jitter, shimmer and Glottal-to-Noise Excitation (GNE) ratio and the voice distribution configuration were evaluated. Voice quality was rated according to the GIRBAS (grade, instability, roughness, breathiness, asthenicity, and strain) scale for auditory perceptual analysis.^8^, ^9^ The voice samples were presented to three experienced speech pathologists whose evaluation was performed simultaneously and consensually. Voice handicap assessment was conducted through application of the Voice Handicap Index (VHI) questionnaire.^10^, ^11^

Central trend and variability measurements were used to describe the numerical variables and frequency distribution was applied to the categorical variables. The nonparametric Mann-Whitney *U* test was applied to investigate associations between the numerical variables (measurements) in groups with two categories, whereas the nonparametric Kruskal-Wallis test was used in the groups with three categories. The Fisher’s exact test was used to compare the categorical variables in 2 × 2 tables. A significance level of 5% was adopted for all statistical tests.

## Results

Laryngeal complaints were referred by 13 patients, most frequently dysphonia (13%–72.22%) and vocal fatigue (6%–33.33%). The following changes were observed during laryngostroboscopy: vertical laryngeal tremor (15%–83.3%), open phase closure (9%–50%), arytenoid edema (4%–22.2%), vocal fold bowing (3%–16.7%), and posterior mucus formation (2%–11.1%). The vibratory behavior was exclusively glottic in all cases.

Regarding the auditory perceptual analysis using the GIRBAS scale, the following distribution of the study sample was found according to category assessed, number of individuals, and rating (degree of deviance from normality): Grade (G) (overall impression of dysphonia) ‒ 9 slight and 6 moderate; Instability (I) ‒ 11 slight and 3 moderate; Roughness (R) ‒ 12 slight and 2 moderate; Breathiness (B) ‒ 3 slight and 1 moderate; Asthenicity (A) ‒ 8 slight and 3 moderate; Strain (S) ‒ 12 slight and 1 moderate.

Acoustic voice analysis showed that some patients only presented changes in the shimmer and jitter parameters. However, all averages were normal, including jitter Period Perturbation Quotient (PPQ) ‒ 0.51% and shimmer Extent Perturbation Quotient (EPQ) ‒ 8.57%. The values of F0 and GNE ratio were within the normality standards.

Application of the self-assessment Voice Handicap Index (VHI) tool according to its three domains showed the following averages: Physical (VHI-P) ‒ 10.5, Functional (VHI-F) ‒ 12.4 and Emotional (VHI-E) ‒ 9.0, with a global score (VHI-total) of 32.5.

Association between the diverse demographic variables and phase closure was evaluated, and no statistically significant differences were observed regarding age, time since disease onset and beginning of symptoms, Hoehn and Yahr stage, categories of the GIRBAS scale, presence of tremor, and measures of voice changes (p > 0.05).

Distribution of the self-perceptual voice measures according to phase closure showed higher VHI-P medians among patients with predominance of open phase (median = 14 vs. 4), but with no statistically significant difference (p = 0.091). In contrast, the VHI-E medians were higher among patients with predominance of closed phase (median = 17 vs. 0) with statistically significant difference (p = 0.029).

Associations between time since disease onset, HY stage and VHI-total with Grade (G) were evaluated. Only VHI-total presented a marginally significant difference (p = 0.056), with the highest VHI-total median observed for the category G = 1. VHI-total and HY stage are independent variables, and the Spearman correlation coefficient of 0.1929 (p = 0.443) confirmed the lack of association between them.

## Discussion

Tongue, larynx and pharynx disorders are observed in 70%–92% of patients.^1^ Verbal communication is the main complaint in 30% of PD patients. Speech can be impaired in a characteristic fashion since disease onset in most patients. In this study, laryngeal complaints were reported by 13 of the 18 patients (72.22%). Dysphonia was the most common symptom, followed by vocal fatigue.

Glottic resistance decrease, air flow increase, lower subglottic pressure, and vocal intensity reduction are found in PD.^2^ Vocal changes can be attributed to incomplete glottic closure, decrease in activation of the laryngeal muscles, muscular atrophy or fatigue, tension or vocal fold movement asymmetry, and rigidity of vocal folds or respiratory muscles.^3^, ^12^

Stroboscopy characterized laryngeal disorders in 22 patients with diagnosis of idiopathic PD, with laryngeal vertical tremor observed in 55% since the earlier stages of disease. The most remarkable findings in stroboscopy among these patients were abnormal glottic closure and phase asymmetry.^13^

In this study, some patients presented only changes in shimmer EPQ and jitter PPQ values at the acoustic analyses. The jitter changes occurred mainly under lack of vocal fold vibration control, whereas the shimmer parameter was principally altered in cases of decreased glottic resistance or presence of mass lesions.^14^

In this series, the VHI-E median was high among patients who presented open phase closure in stroboscopy (p = 0.029). The association between VHI-total and dysphonia grade was marginally significant (p = 0.056).

## Conclusions

Dysphonia was referred by 72.22% of the patients, whereas changes during laryngoscopy were verified in 94.44%. The medians of the acoustic voice analysis and the VHI self-assessment questionnaire were unchanged. The median of VHI emotional domain was higher among patients who presented predominance of open phase closure. There was association between the global VHI score and dysphonia grade.

Financial support

None.

## Declaration of competing interest

The authors declare no conflicts of interest.
